# Synergistic effects of noradrenergic modulation with atomoxetine and 10 Hz repetitive transcranial magnetic stimulation on motor learning in healthy humans

**DOI:** 10.1186/1471-2202-15-46

**Published:** 2014-04-02

**Authors:** Matthias Sczesny-Kaiser, Alica Bauknecht, Oliver Höffken, Martin Tegenthoff, Hubert R Dinse, Dirk Jancke, Klaus Funke, Peter Schwenkreis

**Affiliations:** 1 Department of Neurology, BG-Universitaetsklinikum Bergmannsheil Bochum, Buerkle-de-la-Camp-Platz 1, 44789 Bochum, Germany; 2 Institute for Neuroinformatics, Ruhr-University Bochum, 44780 Bochum, Germany; 3 Department of Neurophysiology, Medical Faculty, Institute of Physiology, Ruhr-University Bochum, 44780 Bochum, Germany

**Keywords:** rTMS, Neuromodulation, Norepinephrine, Atomoxetine, Plasticity, Motor cortex

## Abstract

**Background:**

Repetitive transcranial magnetic stimulation (rTMS) is able to induce changes in neuronal activity that outlast stimulation. The underlying mechanisms are not completely understood. They might be analogous to long-term potentiation or depression, as the duration of the effects seems to implicate changes in synaptic plasticity. Norepinephrine (NE) has been shown to play a crucial role in neuronal plasticity in the healthy and injured human brain. Atomoxetine (ATX) and other NE reuptake inhibitors have been shown to increase excitability in different systems and to influence learning processes. Thus, the combination of two facilitative interventions may lead to further increase in excitability and motor learning. But in some cases homeostatic metaplasticity might protect the brain from harmful hyperexcitability. In this study, the combination of 60 mg ATX and 10 Hz rTMS over the primary motor cortex was used to examine changes in cortical excitability and motor learning and to investigate their influence on synaptic plasticity mechanisms.

**Results:**

The results of this double-blind placebo-controlled study showed that ATX facilitated corticospinal and intracortical excitability in motor cortex. 10 Hertz rTMS applied during a motor task was able to further increase intracortical excitability only in combination with ATX. In addition, only the combination of 10 Hz rTMS and ATX was capable of enhancing the total number of correct responses and reaction time significantly, indicating an interaction effect between rTMS and ATX without signs of homeostatic metaplasticity.

**Conclusion:**

These results suggest that pharmacologically enhanced NE transmission and 10 Hz rTMS exert a synergistic effect on motor cortex excitability and motor learning in healthy humans.

## Background

Repetitive transcranial magnetic stimulation (rTMS) is a non-invasive tool for brain stimulation and is able to modulate brain activity beyond stimulation [1,2]. The mechanisms underlying these long-term rTMS-effects could be analogous to long-term potentiation (LTP) or depression (LTD). These rTMS-induced changes in cortical excitability and brain activity can be measured by different TMS-protocols. Furthermore, rTMS is capable to influence task performance and learning processes. For example, it can improve motor learning [3]. The induced effects depends on different parameters like coil orientation, total number of pulses and frequency.

For the measurement of rTMS-induced changes of cortical excitability, a single-pulse TMS (spTMS) protocol called “stimulus response curve” (SRC) is used. It tests stimulus intensity-dependent recruitment of corticospinal projections by means of spTMS [4]. The steepness of the linear regression line through the data points of the SRC is a measure of corticospinal excitability [5]. Special paired-pulse TMS (ppTMS) protocols can determine intracortical facilitation (ICF) and short-latency intracortical inhibition (SICI) [6]. The normalized ICF and SICI ratios give information about the activity of excitatory and inhibitory intracortical interneuronal circuits [7].

Despite clear effects of rTMS on cortical excitability, identifying consistent effects of rTMS on sensorimotor learning has proven more difficult. Many experiments have found no changes in motor learning after high frequency rTMS in healthy humans, while others showed only mild effects [8,9]. Short lasting improvements, however, could be elicited using a combination of finger tapping task and 10 Hz rTMS [10].

Under these difficult circumstances and to get insight of the physiology of learning processes, numerous studies have used pharmacological interventions [11]. There are several studies on the effect of the positive allosteric modulators of the GABA_A_ receptors, i. e. benzodiazepines [12,13]. For example, Di Lazzaro and coworkers investigated the influence of lorazepam on the excitability on human motor cortex. They could demonstrate by means of single and paired-pulse TMS that lorazepam depress high-amplitude motor-evoked potentials (MEP) and increases the excitability of inhibitory circuits [13]. Moreover, it could be demonstrated that this interference with the GABA_A_-system can reduce learning and use-dependent plastic changes [14]. Similar changes in excitability could be demonstrated by Schwenkreis and coworkers with the glutamate antagonist riluzole and the NMDA antagonist memantine [15,16]. Both agents reduce intracortical facilitation and increase intracortical inhibition. Similar to the observations of Butefisch for lorazepam, it was demonstrated that riluzole and memantine were capable to block use-dependent plasticity in motor cortex.

Looking for pharmacological interventions that lead to facilitative effects and might boost learning processes, the influence of norepinephrine (NE) agonists like amphetamine (AMP), methyphenidate (MPH), reboxetine (RBX) and atomoxetine (ATX) were investigated. MPH, RBX and ATX increase ICF and decrease SICI measured by paired-pulse TMS after a single dosage [8,17,18]. Moreover, Plewnia and Tegenthoff investigated the modulation of use-dependent plasticity in primary motor cortex (M1). RBX and AMP were able to enhance training-induced motor cortex plasticity [8,19]. All these studies clearly show that NE plays a crucial role in promoting plasticity. Especially, ATX affects the regulation of NE as a highly selective inhibitor of the presynaptic NE transporter with low affinity for other transmitters [20,21] whereas AMP and MPH act as indirect NE and dopamine agonists.

Considering these facts, a combination of rTMS and ATX would be a promising intervention that might lead to clear learning effects. So far, there are no placebo-controlled studies on the influence of ATX and rTMS on cortical excitability and motor learning. Moreover, it has not been investigated if a subsequent high frequency rTMS can further increase excitability and motor learning. Here, we used a sequential 4-finger tapping/10 Hz rTMS combination paradigm previously introduced by Kim and coworkers [10] combined with ATX or placebo intake in order to evaluate the effect of atomoxetine and rTMS on motor cortex plasticity and motor learning in humans. Previous studies generally used neuropharmacological modulation only. Here, we were particularly interested in possible interactional effects of both treatment regimens. We therefore administered placebo or ATX and real rTMS or sham rTMS in a randomized double-blind study. We hypothesized that the combination of ATX and 10 Hz rTMS over M1 is capable of increasing excitability and motor learning, and that they might have synergistic effects.

## Results

### Participants

There were no significant differences in sex (p = 0.26) or age (mean ATX + real rTMS group: 24.1 ± 3.95 years, placebo + real rTMS group: 24.6 ± 1.74 years, ATX + sham rTMS group: 24.2 ± 1.98 years, placebo + sham rTMS group: 23.9 ± 2.47 years; F_(3,32)_ = 0.1, p = 0.96). Blood samples were taken from all participants approximately 1 hour and 2 hours after ATX or placebo (PLC) intake. The average ATX blood serum levels in both ATX-groups were 366.1 ng/ml after 1 hour and 296.7 ng/ml after 2 hours after drug intake. Four subjects reported about temporary headache beginning 12 hours after the experiment and persisted about 2 to 4 hours. We could not distinguish if these symptoms derived from ATX or rTMS-intervention.

### Excitability measurements

Considering the slopes of the SRCs, repeated measurement analysis of variance (rmANOVA) showed a significant effect of the within-subject factor “time” (F_(1,2)_ = 19.26, p < 0.000). There was no effect of the between-subject factor “group” (F_(3,32)_ = 1.99, p = 0.14) or the interaction “time x group” (F_(3,6)_ = 0.95, p = 0.47). Post-hoc paired *t*-tests revealed significantly increased slopes from T1 (baseline measurements) to T2 (measurements 1 hour after ATX/placebo intake), from T2 to T3 (measurements after motor task/rTMS combination) and from T1 to T3 (mean slope difference_ΔT2T1_ = 0.006 ± 0.01, t(35) = 2.96, p = 0.005; mean slope difference_ΔT3T2_ = 0.01 ± 0.016, t(35) = 3.7, p = 0.001; mean slope difference _ΔT3T1_ = 0.016 ± 0.02, t(35) = 5.4, p < 0.001).

**Figure 1:**
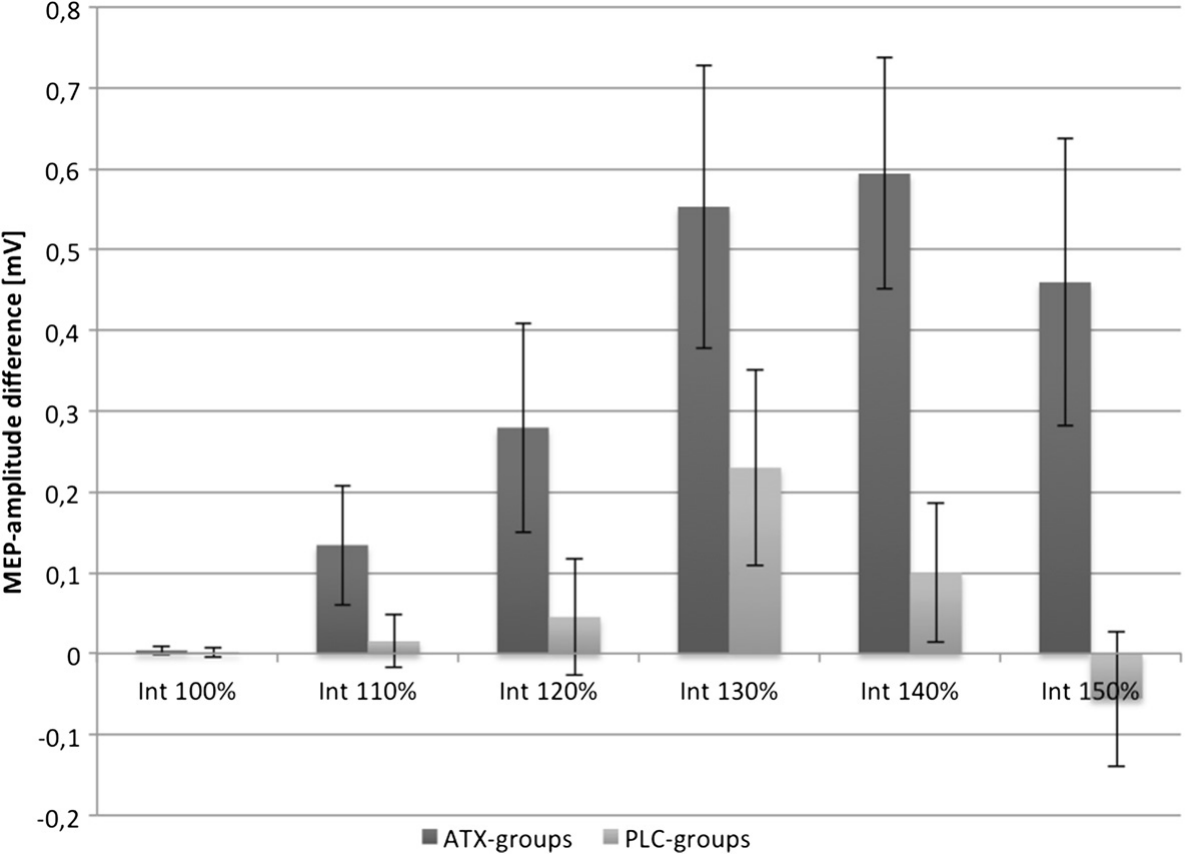
**ATX effects after 1 hour.** Mean-MEP differences of the stimulus-response curves between the measurements T2 and T1. Pooled data of ATX and PLC groups are compared. Error bars depict standard error of the mean.

ATX-induced excitability changes between T1 and T2, being before rTMS intervention, were further assessed by pooling the results of the 2 ATX-groups and the 2 placebo (PLC)-groups. Looking at the slope, rmANOVA with within-subject factor “time” and between-subject factor “group” revealed a significant effect of the factor “time” (F_(1,34)_ = 10.66, p < 0.00), of the interaction between “time” and “group” (F_(1,34)_ = 8.57, p = 0.01) and of the factor “group” (F_(1,34)_ = 5.09, p = 0.03), indicating a greater increase in slope in the ATX-groups as compared to the PLC-groups. For a better overview, Figure 1 shows the mean MEP-differences of ATX- and PLC-groups between T2 and T1 (= **Δ**T2T1) for all 6 stimulation intensities.

Analyzing the SICI-ratio data, rmANOVA revealed a significant effect of the within-subject factor “time” (F_(1,2)_ = 6.69, p < 0.00), no significant effect of the between-subject factor “group” (F_(3,32)_ = 1.14, p = 0.35) and no interaction between “time x group” (F_(3,6)_ = 0.49, p = 0.81). Post-hoc *t*-test showed a significant difference between T1 and T3 (meanSICI-ratio difference_∆T3T1_ = 0.138 ± 0.238, t(35) = 3.5, p = 0.001). The other comparisons were not significant (T1 vs. T2: t(35) = 2.1, p = 0.047; T2 vs. T3: t(35) = 1.9, p = 0.061).

**Figure 2:**
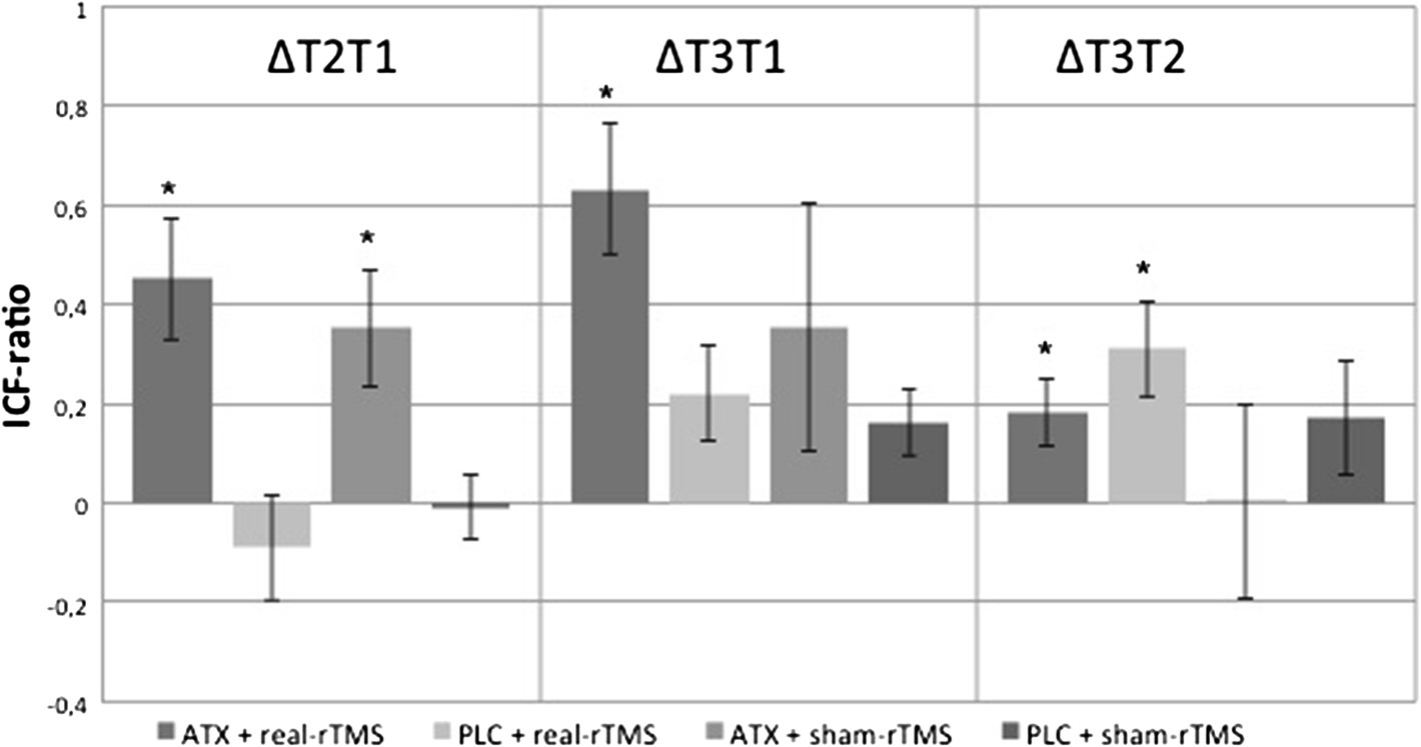
**Intracortical facilitation.** Treatment with 60 mg ATX significantly increased ICF after 1 hour (∆T2T1). Further increase in ICF after rTMS/motor task sequence (∆T3T1). p-values are shown in Table 1 (p – threshold = 0.025). Error bars depict standard error of the mean. The absolute mean ICF-ratio values T2 from T1, T3 from T1 and T3 from T2 were subtracted (mean ICF-ratio difference _∆T2T1,_ mean ICF-ratio difference _∆T3T1_, mean ICF-ratio difference _∆T3T1_) to calculate the absolute difference. * = significant differences.

RmANOVA of the ICF-ratio data showed a significant effect of the within-subject factor “time” (F_(1,2)_ = 13.9, p < 0.001). Furthermore, a significant interaction between the factor “time” and the between-subject factor “group” could be demonstrated (F_(3,6)_ = 2.57, p = 0.03). The between-subject factor “group” revealed no significant effect (F_(3,32)_ = 2.35, p = 0.09). Post-hoc *t*-test showed a significant increase of the ICF-ratio 1 hour after drug intake in both ATX-groups (∆T2T1). ICF-ratio increased further in the ATX + real-rTMS-group after motor training and 10 Hz rTMS (∆T3T2). This could not be demonstrated for the ATX + sham-rTMS-group. Mean ICF-ratio differences_∆T2T1_, _∆T3T1_ and _∆T3T2_ and corresponding p-values are shown in Table 1 and Figure 2.

**Table 1:** ICF-ratio data

**Groups**	**∆T2T1**	**∆T2T1**	**∆T3T1**	**∆T3T1**	**∆T3T2**	**∆T3T2**
	**Mean ICF-ratio**	**t-value**	**Mean ICF-ratio**	**t-value**	**Mean ICF-ratio**	**t-value**
	**difference ± SEM**	**p-value**	**difference ± SEM**	**p-value**	**difference ± SEM**	**p-value**
ATX + real-rTMS	0.451 ± 0.12	t(8) = 3.7	0.632 ± 0.13	t(8) = 4.8	0.181 ± 0.07	t(8) = 2.7
		*0.006		*0.001		*0.025
PLC + real-rTMS	-0.089 ± 0.11	t(8) = -0.8	0.221 ± 0.1	t(8) = 2.3	0.311 ± 0.1	t(8) = 3.2
		0.425		0.052		*0.012
ATX + sham-rTMS	0.352 ± 0.12	t(8) = 2.97	0.354 ± 0.25	t(8) = 1.4	0.002 ± 0.19	t(8) = -0.01
		*0.018		0.194		0.99
PLC + sham-rTMS	-0.009 ± 0.07	t(8) = -0.1	0.163 ± 0.07	t(8) = 2.4	0.172 ± 0.12	t(8) = 1.5
		0.9		0.043		0.17

Multiple paired two-sided post-hoc *t*-test of ICF-ratio data. Bonferroni-corrected p-value threshold (p = 0.025). *significant.

No effect of either ATX or rTMS could be shown for RMT (within-subject factor “time”: F_(1,2)_ = 1.22, p = 0.3; between-subject factor “group”: F_(3,32)_ = 1.78, p = 0.17; interaction “time x group”: F_(3,6)_ = 0.66, p = 0.68).

### Behavioral data

**Figure 3:**
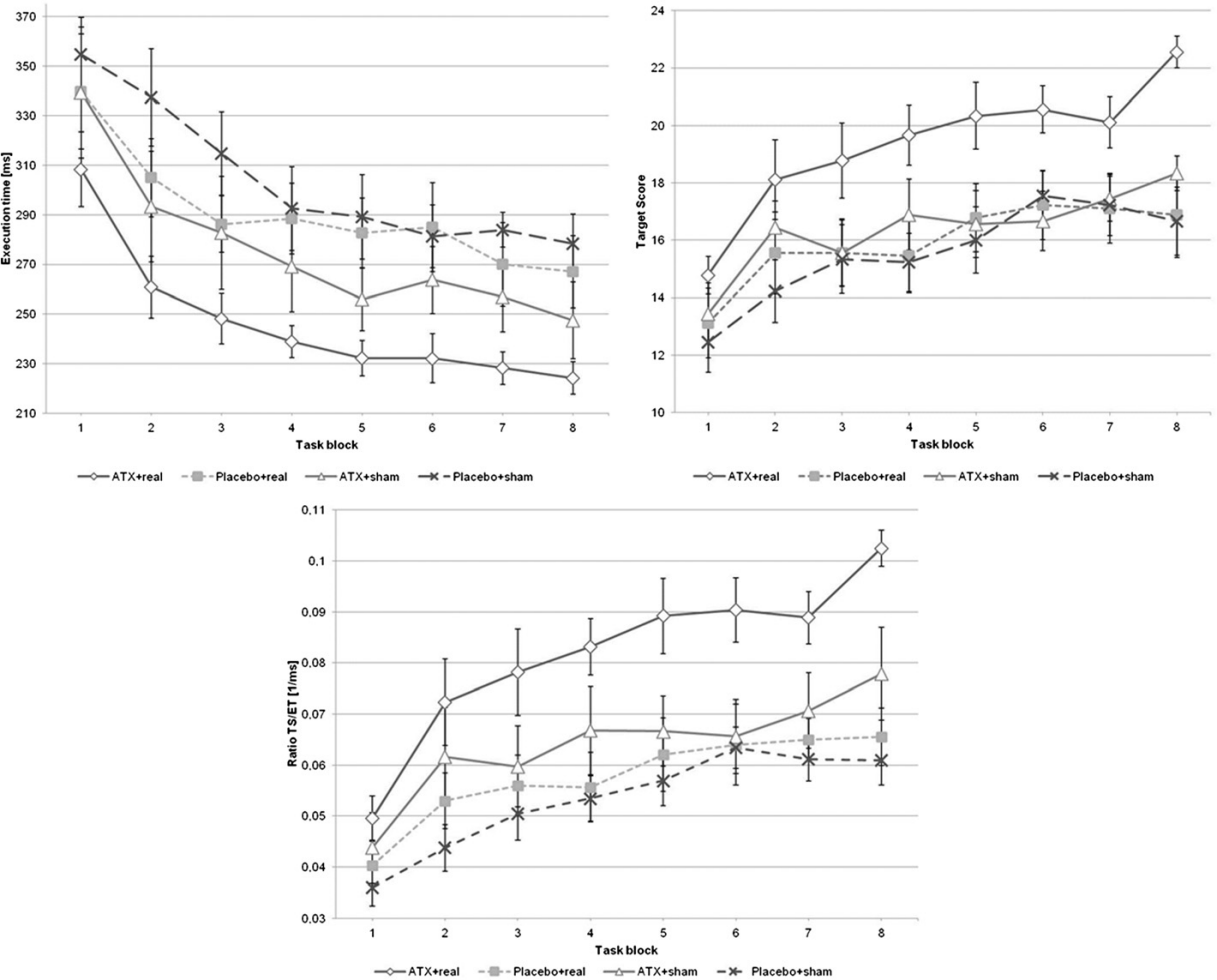
**Motor task data.** Time course of target score (TS), execution time (ET) and TS/ET-ratio (TSET) is shown for each group. The motor task consisted of 8 blocks. Error bars indicate standard error of the mean.

Analyzing target-score (TS) data, rmANOVA demonstrated a significant effect of the within-subject factor “time” and the between-subject factor “group” (F_(1,7)_ = 21.02, p < 0.001; F_(3,32)_ = 3.79, p = 0.02) but no significant interaction “time x group” (F_(3,21)_ = 0.93, p = 0.56; see Figure 3. Post-hoc independent two-sided *t*-tests revealed a significant difference between PLC + sham-rTMS and ATX + real-rTMS group (t(16) = 3.1, p = 0.007), but not for PLC + sham-rTMS and ATX + sham-rTMS (t(16) = 0.6, p = 0.563) and PLC + sham-rTMS and PLC + real-rTMS (t(16) = 0.3, p = 0.754). Similar results could be shown for the execution-time (ET)- and TSET ratio (= ratio of target score and execution time)-analyses (see Figure 3). Considering ET, rmANOVA showed significant effects for the within-subject factor “time” (F_(1,7)_ = 47.33, p < 0.001) and the between-subject factor “group” (F_(3,32)_ = 3.3, p = 0.03) without a significant interaction between both factors (F_(3,21)_ = 0.65, p = 0.87). Post-hoc independent two-sided *t*-tests demonstrated significant differences only between the PLC + sham-rTMS and ATX + real-rTMS group (t(16) = -3.9, p = 0.001). RmANOVA of the TSET ratio demonstrated a significant influence of the between-subject factor “group” (F_(3,32)_ = 5.13, p = 0.01) and the within-subject factor “time” (F_(1,7)_ = 39.14, p < 0.001). There was no significant interaction “time x group” (F_(3,21)_ = 1.53, p = 0.07; see Figure 3). Again, post hoc *t*-test showed significant higher TSET ratio for the ATX + real-rTMS group compared to the PLC + sham-rTMS group (t(16) = 4.4, p < 0.001). There were no significant differences between PLC + sham-rTMS group and PLC + real-rTMS or ATX + sham-rTMS group. No significant differences could be shown for the ER (rmANOVA: within-subject factor “time”: F_(1,7)_ = 0.01, p = 0.24; between-subject factor “group”: F_(3,32)_ = 1.11, p = 0.36; interaction “time x group”: F_(3,21)_ = 0.71, p = 0.82).

## Discussion

Our study yielded three major findings. First, ATX led to a significant increase of corticospinal and intracortical excitability in M1 one hour after intake of 60 mg ATX (see Figures 1 and 2). Second, high frequency 10 Hz rTMS applied over M1 during a finger tapping motor task was capable of further increasing intracortical excitability significantly, but only in combination with 60 mg ATX (see Figure 2). Third, only the combination of ATX and 10 Hz rTMS significantly improved motor learning with regard to target score and execution time (see Figure 3).

### ATX led to a significant increase of corticospinal and intracortical excitability in M1

We could reproduce the facilitative effects of ATX on cortical excitability in M1 as had been previously shown by *Gilbert* and coworkers [18]*.* In contrast, we did not see a significant ATX-induced M1 disinhibition. The reduction of intracortical inhibition between the beginning and end of the study (∆T3T1) did not depend on the group, i.e. on the type of intervention. Similar effects of NE on M1 excitability could be demonstrated for the NE reuptake inhibitor reboxetine. *Plewnia* and coworkers [17,19,22] showed enhanced corticospinal and intracortical excitability and improved motor skills in healthy subjects suggesting that this is an effect of NE reuptake inhibitors. This assumption could not be verified by *Foster* and coworkers [23]. They found no improvement of motor learning after intake of the NE reuptake inhibitor venlafaxine compared to ATX [23]. They concluded that the affinity to other transmitters like serotonin and the lower dosage and the higher rate of adverse effects of venlafaxine might have led to contradictory results.

### High frequency 10 Hz rTMS applied over M1 during a finger tapping motor task was capable of further increasing intracortical excitability significantly, but only in combination with ATX

In our study, we wanted to extend the approach of neuropharmacological modulation of cortical activity and its use-dependent plasticity by additionally applying 10 Hz rTMS. Our rTMS paradigm itself had no significant facilitative effects on excitability parameters. This might be due to the low number of total TMS-pulses (i. e. 160 pulses). It is well known that rTMS effects depend on the number of total pulses, frequency and stimulation intensity [3]. Interestingly, we could see a further increase in excitability only in combination with 60 mg ATX (ATX + real-rTMS group). This suggests that a premedication with ATX is capable of facilitating the effects of a low pulse number rTMS protocol. Homeostatic plasticity did not play a role in this study. Following the concept of homeostatic plasticity, we would have expected that the enhancement of motor cortex excitability induced by ATX favors the induction of synaptic depression by the subsequent 10 Hz rTMS-stimulation and motor task that themselves would induce LTP-like plasticity. Cortical LTP and LTD are typically mediated by NMDA-receptor activation [24]. One reason, why we could not see homeoplastic effects is that our 1^st^ intervention (ATX intake) had no effect on NMDA-receptors but on NE-receptors. All classic homeostatic plasticity protocols combine rTMS, transcranial direct current stimulation (tDCS) or paired-associative stimulation protocols that typically involves NMDA-receptors [25,26], for example 1 Hz rTMS with cathodal tDCS [27].

Instead of homeostatic effects, a synergistic effect of ATX and rTMS was observed. The sum of gain in excitability in the ATX + 10 Hz rTMS group could not be explained by the single effects of ATX and 10 Hz rTMS. Because higher cortical excitability is a precondition for neuronal plasticity and improved learning process, this finding might be closely related to the fact that we observed improved motor learning only after combining both interventions. The significant increase in ICF-ratio in the PLC + sham-rTMS condition could be explained by the motor task itself [28].

### Only the combination of ATX and 10 Hz rTMS improved motor learning and execution time significantly

Looking at the motor task data, significant higher TS and TSET ratio and shorter ET could only be seen in the verum-verum-condition, i. e. ATX + real-rTMS group. There was no significant interaction between time and group. Graphically, the TS, ET and TSET curves are shifted in a parallel fashion. This indicates that the superiority of the ATX + real-rTMS group was not based upon a different effect on motor learning itself but upon a better performance at the beginning of the motor task due to higher cortical excitability as previously mentioned. Another reason might be the well known ATX effects in promoting wakening and arousal [29]. In contrast to the results of *Kim* and coworkers [10], we did not observe higher TS for the PLC + real-rTMS group compared to the PLC + sham-rTMS. This may be due to our modified and easier motor task, which could have prevented smaller differences in learning to become visible.

Considering detail of our motor task, we decided to choose the non-dominant hand and according non-dominant hemisphere (left hand, right hemisphere) for motor task, excitability measurements and rTMS-intervention because we wanted to avoid a ceiling effect [10]. We did not test the excitability parameters of the contralateral hemisphere and motor learning of the contralateral hand. Thus, previous studies could demonstrate that there is a hemispheric asymmetry of corticospinal activation with a higher MEP facilitation for the non-dominant (left) hemisphere [30,31]. Brouwer et al. showed that this different level of excitation is not related to speed or dexterity of finger movements. Relating to our study, we would expect similar facilitative effects of ATX and rTMS on dominant hemisphere. We also can purpose that there were differences in levels of cortical excitability between hemispheres not only in baseline but also later in postinterventional measurements. But according to Brouwer’s results, these differences had no relation to differences in motor performance between both hands.

**Figure 4:**
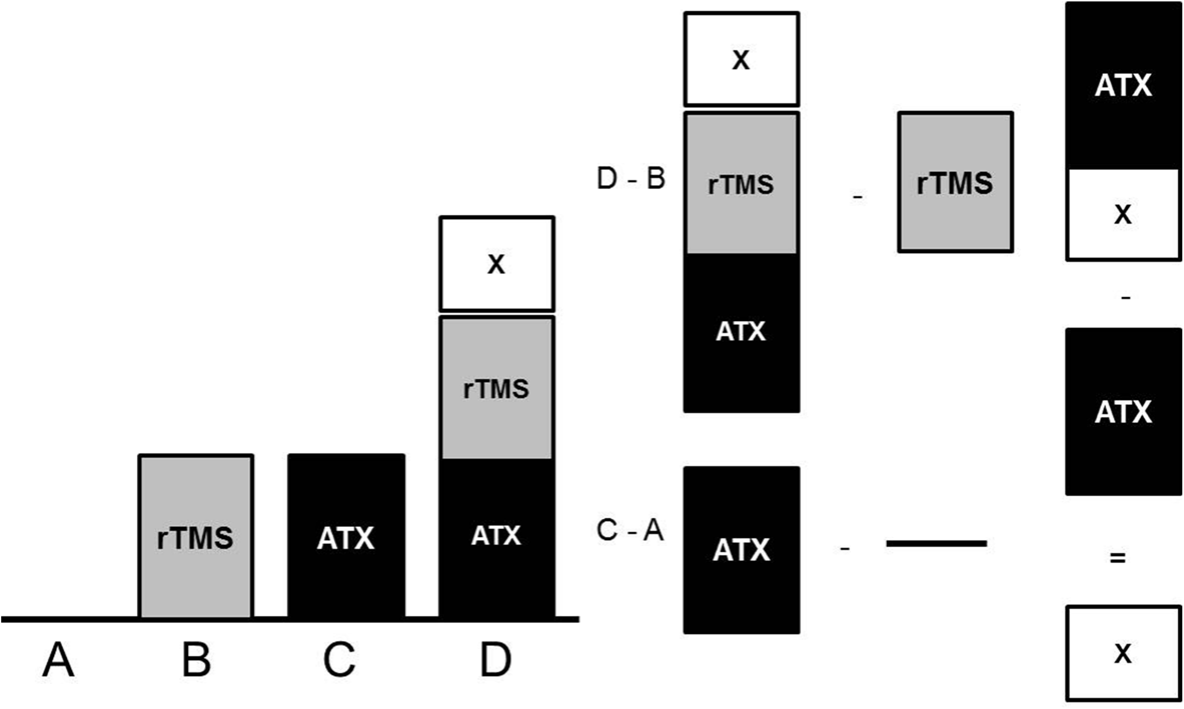
**Factorial design.** This graphic shows the idea of a synergistic effect that only occurs in the verum-verum-group D (combination of both interventions). Furthermore, it shows how to calculate the synergistic effect. Group A = PLC + sham-rTMS; group B = PLC + real-rTMS; group C = ATX + sham-rTMS; group D = ATX + real-rTMS. X indicates interaction factor “ATX x real-rTMS” (synergistic effect).

Interestingly, motor learning was not affected by ATX or 10 Hz rTMS alone. According to the *subtraction method* devised by *Donders*[32], the entire effects on TS or ET should be explained by the cooperativity of the ATX and rTMS effects. Applying this method, a factor “X” remains which cannot be explained by the separate ATX and rTMS effects (see Figure 4). This kind of *synergistic effect* “ATX x rTMS” indicates nonlinear interaction between both components. The neuronal basis of this interaction, however, remains unclear. High frequency rTMS activates the motor network including premotor cortex, supplemental motor area and ipsilateral primary motor cortex, and increases effective connectivity between these areas [33,34]. The finger tapping task itself activates a similar system including contralateral primary sensorimotor cortex, premotor cortex, supplementary motor cortex, bilateral secondary somatosensory areas and basal ganglia and ipsilateral cerebellum [35]. In addition, ATX leads to significant increase of activity in error signaling forebrain areas like bilateral inferior frontal cortex and pre-supplementary motor cortex, increasing neural sensitivity for errors in healthy humans [36].

Therefore, the combination of more effective synaptic transmission within the motor system along with higher cognitive/behavioral sensitivity may have led to the synergistic effect of 10 Hz rTMS and ATX seen in our study. It could also explain the failure of ATX or 10 Hz rTMS alone to be effective.

### Pharmacological interventions and rTMS

Studies combining rTMS and neuropharmacological intervention were usually undertaken to investigate the role of transmitters in the induction of rTMS after-effects and not to boost performance like we did in our study. Huang et al. combined a specialized rTMS-protocol called theta-burst-stimulation (TBS) with the NMDA receptor antagonist memantine [25]. They found that, on one hand, memantine inhibited the facilitatory effect of intermittent TBS (iTBS) on MEP amplitudes. On the other hand, it blocked the suppressive effect of continuous (cTBS). Teo et al. used the NMDA receptor coagonist D-cycloserine that acts at the glycine site of the NMDA receptor. They found that it reversed the aftereffect of iTBS from facilitation to inhibition [26]. Lang et al. performed a 1 Hz rTMS study using the dopamine receptor agonist pergolide and found that the suppression of corticospinal excitability by rTMS was more pronounced after drug intake compared to placebo [37]. These results show in general that NMDA and dopaminergic receptors play a role in the induction of rTMS effects.

So far, no study has been undertaken to investigate adrenergic influences on rTMS effects. Furthermore, we have not found any study that considers the combination of pharmacological intervention, rTMS and motor learning. In this case, we describe synergistic effects between rTMS and pharmacological modulation for the first time.

### Limitations

Considering some limitations of our study, we surely have to mention the relative low number of subject per group (n = 9). Furthermore, we did not choose a cross-over design, which would have allowed intra-subjects comparison. A cross-over design would have had advantageous for the interpretation of the results, rendering out biases that comes from inter-individual variability of cortical excitability, partly determined by brain morphology [38,39].

Moreover, one might argue that the use of circular coil for excitability measurement does not extract reliable measures compared to a figure-of-eight coil. Ugawa et al. could demonstrate no different results between figure-of-eight coil over left hand motor area and circular coil over vertex for the determination of corticocortical facilitation and inhibition [40]. Moreover, in our TMS-studies, we prefer the use of the circular coil. We could see stable results and the positioning of this coil is less critical [41-43].

## Conclusion

Previous studies could show that high frequency rTMS and ATX are capable to modulate cortical plasticity and to improve motor learning [8,9]. Possible interaction effects have never been investigated. In the present study, we could show that the combination of a pharmacologically-induced increase in NE transmission and 10 Hz rTMS exerts synergistic effects on cortical excitability and motor learning in healthy humans.

This could be a promising approach to improve motor learning in patients with neurological disorders like stroke, traumatic brain injury and neuromuscular diseases (e. g. amyotrophic lateral sclerosis). Especially, it would be interesting to investigate the development and consolidation of neuronal plasticity effects in primary motor cortex when rTMS and ATX were administered over several days. Furthermore, it remains unclear if such effects would be seen with other facilitative drugs like modafinil or amantadine or in combination with other brain stimulation protocols like tDCS and TBS.

## Methods

### Subjects

Data from 36 healthy subjects (19 women, 17 men) were collected and analyzed. Subjects were randomly assigned to four equally-sized groups (n = 9). All subjects gave their written informed consent. The protocol was approved by the local ethical committee of the Ruhr-University of Bochum (registry no. 4317-12) and was performed in accordance with the Declaration of Helsinki. The study is registered in German Clinical Trials Register (DRKS-ID: DRKS00004653). All subjects were right-handed as revealed by the Edinburgh Handedness Inventory [44]. They all denied the practice of fine motor skills presently or in the past, such as playing a guitar or piano or as having experience in professional typewriting. All participants were free of medication.

### Time course and study design

**Figure 5:**
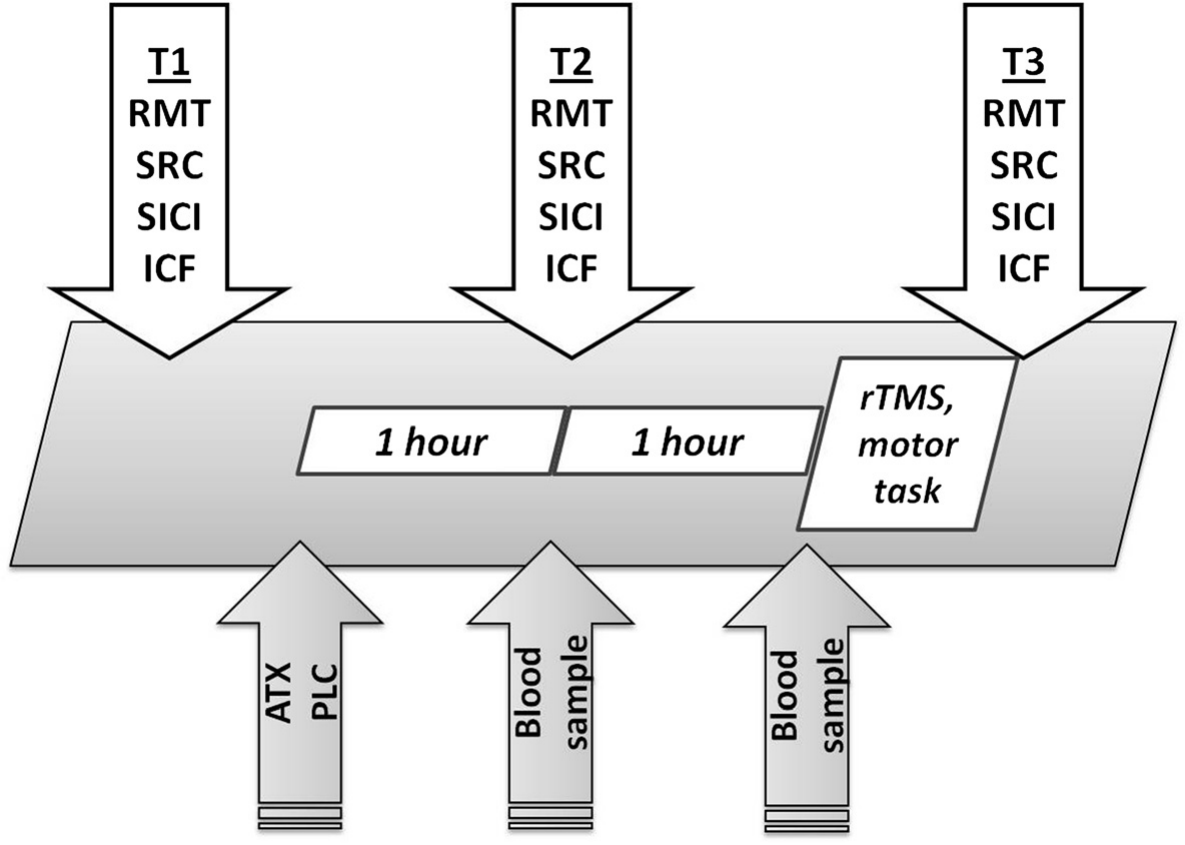
Time course of the study and study design.

The study was randomized and placebo-controlled. It was double-blind for the ATX vs. PLC condition and single-blind for the rTMS and sham-rTMS condition. A single dose of 60 mg ATX or placebo was given after baseline excitability measurements (T1). Blood samples were collected one and two hours after administration of ATX or PLC to determine ATX plasma concentration. One hour after drug intake, excitability measurements were repeated (T2). Then, all participants performed a sequential motor task with their non-dominant left hand while intermittently receiving single-blinded real 10 Hz rTMS or sham-rTMS in the manner described below. Finally, excitability parameters were measured again (T3). Each measurement session took approximately 30 to 40 minutes, the rTMS + motor practice 10 minutes, and the entire study three hours (see Figure 5).

### Substances

Participants left TMS-laboratory after baseline measurements and went to a special room where the non-blinded examiner (P.S.) who was participated in randomization and administration of the drugs only delivered the capsules. Subjects received either a yellow-blue 60 mg ATX capsule (Eli Lilly™) or a yellow-red capsule with mannitol (placebo). They swallowed it with a drink of water in this room and stayed for one hour before returning in TMS-laboratory. The TMS-examiners have seen neither the capsules nor the drug intake itself. The subjects and TMS-examiner did not know color code.

### Excitability measurements by TMS

The following parameters of corticospinal and intracortical excitability in the primary motor cortex were investigated: resting motor threshold (RMT), stimulus-response curve [4] and both short intracortical inhibition and intracortical facilitation, assessed using paired-pulse TMS [6]. MEPs were recorded with Ag-AgCl-surface electrodes using a standard electromyography device (Neuropack M1; Nihon Kohden, Tokyo, Japan). While stimulating the contralateral hemisphere, recordings were taken from the left first dorsal interosseus muscle (FDI). The signals were recorded with a sampling rate of 5 kHz, and amplified with a bandpass of 20 Hz - 3 kHz, a sweep duration of 10 - 50 ms/div and a gain of 0.1 mV/div. During the entire measurements, muscle relaxation was monitored by EMG. Subjects were seated in a comfortable chair in a silent and bright room.

#### Resting motor threshold

RMT was determined with single-pulse TMS to the nearest 1% of the stimulator output, and was defined as the minimum intensity which produced four motor evoked potentials > 50 μV out of eight trials [45]. Single-pulse TMS was applied using a Magstim 200® stimulator (Magstim, Whitland, Dyfed, U.K.) connected to a circular coil (outer diameter 14 cm). The coil was placed with its center near the vertex with the current flowing clockwise in the coil in order to activate predominantly the right hemisphere and to produce MEP in the left FDI. This position was marked with a red wax pencil to improve reproducibility of placement.

#### Stimulus-response curve

For SRC, spTMS was applied at 100%, 110%, 120%, 130%, 140% and 150% of individual RMT. For each stimulus intensity, 12 trials were performed. We used the same TMS-setting for SRC as for RMT-determination. For analysis of the SRC data, the slope between data points T_*i, i =1, 2, 3*_ of the SRC as the steepness of the linear regression line through the given data points (between 100% and 150% stimulation intensity of the individual RMT) was calculated [5].

#### Paired-pulse TMS

To apply ppTMS, the circular coil was connected to the Bistim® device which triggered two Magstim 200® stimulators. Earlier studies had shown that focal and circular coils elicited comparable results in pp-TMS studies [40]. In this ppTMS assembly, a separate RMT had to be determined because of the lowered stimulator output in case the Magstim 200 is connected to the Bistim® device. We tested the interstimulus intervals (ISI) 2, 4, 10 and 15 ms. The second stimulus (test stimulus) was adjusted to evoke an MEP of approximately 1.0 mV. The conditioning stimulus was set at 80% of the individual ppTMS assembly RMT. For each ISI, 12 trials were performed. Before and after ppTMS, 12 single control stimuli using the same stimulation intensity as for the second (test) stimulus were applied. The amplitude ratio of the mean conditioned MEP to the mean control MEP was calculated for each ISI. For further statistical analysis, parameters of SICI and ICF were defined as the averages of MEP ratios obtained at inhibitory ISIs of 2 and 4 ms (SICI), and at facilitative ISIs of 10 and 15 ms (ICF) [41,46]. ICF-ratios between the three time points were compared by subtracting the means (mean ICF-ratio difference_∆T2T1,_ mean ICF-ratio difference_∆T3T1_ and mean ICF-ratio difference_∆T3T2_).

### Motor task

**Figure 6:**
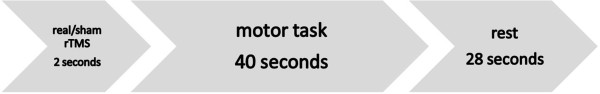
Design of rTMS/motor-task sequence.

A modified combined session of motor task and 10 Hz rTMS as previously described by *Kim* and coworkers [10] was used. The task was designed using Presentation® software (Neurobehavioral Systems Inc., Albany, California, USA). Subjects were seated in a comfortable chair and placed 35 cm in front of a 19 inch LED-monitor. A seven-digit sequence of numbers, namely the combination of *“1 2 3 4 1 2 3”* was presented on the monitor for 40 seconds. Subjects were instructed to repeatedly push the four numbered buttons of the response box (Lumitouch™, Photon Control, Burnaby, Canada) as *accurately, as quickly* and as often as possible using their left hand (one task block). In consideration of this quite easy tapping task, we decided to choose the non-dominant and non-specialized left hand to ensure motor improvement and learning and to avoid an early ceiling effect. Our decision was not based on electrophysiological aspects like asymmetry in corticospinal activation [30] but on motor task aspects. Each button relates to one finger (no. 1 to the index finger, no. 2 to the middle finger, no. 3 to the ring finger, and no. 4 to the little finger). The motor task required the repetition of eight identical task blocks with pauses of 28 seconds interleaved (see Figure 6). Within each task block, motor practice was preceded by rTMS application. Before starting the entire motor task, subjects were allowed to perform two blocks of practice to familiarize themselves with the equipment and experimental procedure. In this case, we used a different combination of numbers.

### rTMS

For rTMS application, a Magstim Rapid® stimulator (Magstim, Whitland, Dyfed, UK) and a figure-of-eight shaped coil (outside diameter 8.7 cm, peak magnetic field strength 2.2 T, peak electric field strength 660 V/m) were used. The coil predominantly stimulates neural structures below the junction of the two coils. During the entire stimulation procedure the coil was held tangentially to the head in posterior-anterior direction with the handle pointing backwards.

Real- and sham-rTMS were delivered over the right motor cortex at the scalp position where suprathreshold spTMS elicited the highest MEP amplitude (hotspot of the FDI). At the FDI hotspot, we had to determine a new RMT because we used a figure-of-eight coil for focal rTMS. Twenty pulses of 10 Hz rTMS were applied for 2 seconds just prior to the beginning of each task block with an intensity of 80% of individual RMT. A total of 160 pulses were given during each experiment consisting of eight task blocks. For sham-rTMS, the same stimulation parameters and the same figure-of-eight-coil were used, except for the stimulation intensity, which was set at the lowest possible stimulation intensity (10% of maximal stimulator output). Previous work of ours confirmed this intensity to have only local effects at the scalp with no effects on neuronal excitability in the motor cortex [9].

### ATX serum concentration

Blood samples were taken from all participants approximately 1 hour and 2 hours after drug or PLC intake. After finishing the study, ATX serum concentration was determined by liquid-chromatography tandem mass spectrometry method (Laboratoriumsmedizin Dr. Eberhard & Co., Dortmund, Germany).

### Statistical analysis

RmANOVA was performed with the within-subject factor “time” and between-subject factor “group” to assess differences between different points of measurement for the excitability parameters. Univariate ANOVA was performed using age as dependent variables and group as the between-subject factor. A chi-squared test was used to analyze sex differences between the different groups. For analysis of the behavioral data, we considered the total number of correct responses of an entire 7-digit sequence (target score = TS), the execution time (ET) defined by the time required to the subsequent correct response, the ratio of TS and ET (TSET) and the error rate (ER) defined by the equation

(1) $$\mathrm{ER}=1-\frac{\mathrm{TS}}{\max\;\mathrm{TS}}.$$

RmANOVA was performed with within-subject factor “time” and between-subject factor “group”. Where it was appropriate, post-hoc two-sided *t*-tests were additionally applied. The significance level was adjusted by dividing it by the number of comparisons (*Bonferroni* correction). All calculations were performed using IBM SPSS Statistics 19.0 software package.

## Abbreviations

ATX: Atomoxetine; ANOVA: Analysis of variance; ET: Execution time; ER: Error rate; NE: Norepinephrine; ppTMS: paired-pulse TMS; rmANOVA: Repeated measures ANOVA; RMT: Resting motor threshold; rTMS: repetitive TMS; SEM: Standard error of the mean; spTMS: single-pulse TMS; SRC: Stimulus-response curve; TMS: Transcranial magnetic stimulation; TS: Target score; TSET: Ratio of TS and ET; uANOVA: univariate ANOVA.

## Competing interests

The authors declare that they have no competing interests.

## Authors’ contributions

MSK, AB, OH, PS, MT, HRD, KF and DJ participated in the design of the study, the discussion of the results and drafted the manuscript. MSK and AB conducted the experiments. MSK, AB, OH and PS performed the statistical analysis. All authors read and approved the final manuscript.
